# The complete chloroplast genome of *Amomum tsao-ko*

**DOI:** 10.1080/23802359.2020.1717382

**Published:** 2020-01-24

**Authors:** Mengli Ma, Bingyue Lu

**Affiliations:** Key Laboratory for Research and Utilization of Characteristic Biological Resources in Southern Yunnan, College of Life Science and Technology, Honghe University, Mengzi, Yunnan, PR China

**Keywords:** *Amomum tsao-ko*, complete chloroplast genome, phylogenetic analysis

## Abstract

*Amomum tsao-ko* (Zingiberaceae) is an important edible and medicinal crop. The complete chloroplast (cp) genome of *A. tsao-ko* was determined using Illumina sequencing platform. The size of whole cp genome was 163,648 bp, containing a small single copy (SSC) region of 15,355 bp and a large single copy (LSC) region of 88,741 bp, which were separated by a pair of inverted repeat (IRs) regions (29,776 bp). The *A. tsao-ko* cp genome contained 133 genes, including eight ribosomal RNA genes (4 rRNA species), 38 transfer RNA genes (30 tRNA species) and 87 protein-coding genes (79 PCG species). The overall GC content of *A. tsao-ko* cp genome is 36.02%. To investigate the evolution status of *A. tsao-ko*, as well as Zingiberales, a phylogenetic tree with *A. tsao-ko* and other 16 species was constructed based on their complete chloroplast genomes. Phylogenetic analysis revealed that *A. tsao-ko* was closely related to *Alpinia zerumbet*.

*Amomum tsao-ko Crevost & Lemarié* belongs to the genus *Amomum* of Zingiberaceae, the fruit is a commonly used traditional Chinese medicine with the effects of dispelling cold and warming spleen as well as eliminating dampness and phlegm, which can also be used as cooking condiments and widely used as flavoring spices for food processing (Lim, 2013; Shi et al. 2014). At present, the research on *A. tsao-ko* mainly focuses on the chemical composition and pharmacological effects, while there are few studies on the molecular aspects (Lu et al. 2018). The complete chloroplast genome would be useful to shed light on the phylogenetic relationships of *A. tsao-ko* (Wu et al. 2017; Li et al. 2019). In this study, the complete chloroplast genome sequence of *A. tsao-ko* is first reported, it will provide useful information for better understanding the evolution of the whole Zingiberaceae family.

Fresh leaves of *A. tsao-ko* were collected from Jinping County (22°54′30.34″N, 103°13′16.39″E), Yunnan Province, China. The voucher specimen (LBY20180526) was deposited in Herbarium of Honghe University, China. Approximately 5 g of fresh leaves was harvested for chloroplast DNA isolation (McPherson et al. 2013). After DNA isolation, purified cp DNA was used for short-insert libraries construction (Borgstrom et al. 2011), the whole cp genome sequencing was conducted by BIOZERON Co., Ltd. (Shanghai, China) on the Illumina Hiseq 4000 platform. Then we used the software SOAPdenovo2.04 to assemble the complete cp genome of *A. tsao-ko* (Luo et al. 2012) and the genes were annotated using an online DOGMA tool (Wyman et al. 2004). Finally, the assembled and annotated chloroplast genome was submitted to GenBank database (accession no. MK926774).

The complete cp genome of the *A. tsao-ko* was 163,648 bp, containing a small single copy (SSC) region of 15,355 bp and a large single copy (LSC) region of 88,741 bp, which were separated by a pair of inverted repeat (IRs) regions (29,776 bp). The *A. tsao-ko* circular cp genome contained 133 genes, including 8 ribosomal RNA genes (4 rRNA species), 38 transfer RNA genes (30 tRNA species) and 87 protein-coding genes (79 PCG species). Most of the gene species occurred in a single copy, while 20 gene species occurred in double copies, including four rRNA species (23S, 16S, 5S and 4.5S rRNA), eight tRNA species (trnA-UGC, trnI-CAU, trnI-GAU, trnH-GUG, trnL-CAA, and trnN-GUU, trnR-ACG, trnV-GAC), and eight PCG species (rps7, rps12, rps19, rpl2, rpl23, ycf1, ycf2 and ndhB). The overall GC content of the circular genome was 36.02%.

To obtain its evolution status of *A. tsao-ko* within the order Zingiberales, the phylogenetic relationships were constructed by complete chloroplast genomes of 17 species (*Xiphidium caeruleum* and *Panax notoginseng* as the outgroup). The alignment was performed using software MAFFT (Katoh and Standley 2013). A maximum likelihood (ML) tree was generated by MEGA6.0 (Tamura et al. 2013) using 1000 bootstrap replicates. The phylogenetic analysis showed that *A. tsao-ko* is closely related to *Alpinia zerumbet* (Figure 1). In summary, we characterized complete chloroplast genome that could provide a better understanding of *A. tsao-ko*, and also provide a reference for identification, protection and utilization of the genus *Amomum* plant resources.

**Figure 1.**
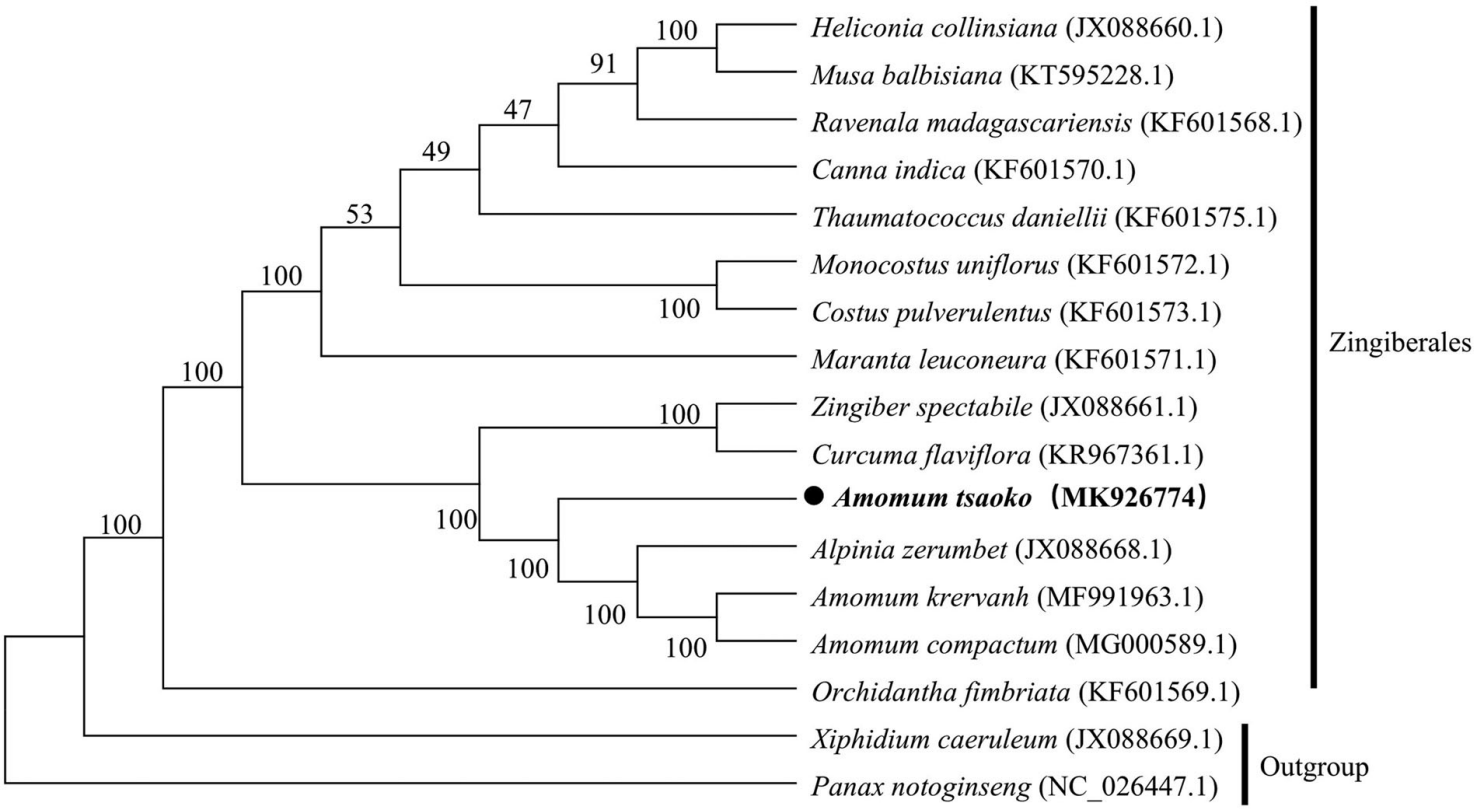
Phylogenetic tree based on the complete chloroplast genome sequences of *A. tsao-ko* and 16 other species (contain 2 outgroup *Xiphidium caeruleum* and *Panax notoginseng*). Numbers on the nodes indicate bootstrap values.
